# EGF regulates tyrosine phosphorylation and membrane-translocation of the scaffold protein Tks5

**DOI:** 10.1186/1750-2187-8-8

**Published:** 2013-08-07

**Authors:** Anna Fekete, Gábor Bőgel, Szabolcs Pesti, Zalán Péterfi, Miklós Geiszt, László Buday

**Affiliations:** 1 Institute of Enzymology, Research Center for Natural Sciences, Hungarian Academy of Sciences, Budapest 1113, Hungary; 2 Department of Medical Chemistry, Semmelweis University Medical School, Budapest 1094, Hungary; 3 Department of Physiology, Semmelweis University Medical School, Budapest, Hungary; 4 “Lendület” Peroxidase Enzyme Research Group of the Semmelweis University and the Hungarian Academy of Sciences, Budapest 1094, Hungary

**Keywords:** EGF receptor, Tks5, Tks4, PX domain, PI 3-kinase, Src

## Abstract

**Background:**

Tks5/FISH is a scaffold protein comprising of five SH3 domains and one PX domain. Tks5 is a substrate of the tyrosine kinase Src and is required for the organization of podosomes/invadopodia implicated in invasion of tumor cells. Recent data have suggested that a close homologue of Tks5, Tks4, is implicated in the EGF signaling.

**Results:**

Here, we report that Tks5 is a component of the EGF signaling pathway. In EGF-treated cells, Tks5 is tyrosine phosphorylated within minutes and the level of phosphorylation is sustained for at least 2 hours. Using specific kinase inhibitors, we demonstrate that tyrosine phosphorylation of Tks5 is catalyzed by Src tyrosine kinase. We show that treatment of cells with EGF results in plasma membrane translocation of Tks5. In addition, treatment of cells with LY294002, an inhibitor of PI 3-kinase, or mutation of the PX domain reduces tyrosine phosphorylation and membrane translocation of Tks5.

**Conclusions:**

Our results identify Tks5 as a novel component of the EGF signaling pathway.

## Background

Epidermal growth factor receptor (EGFR) is involved in diverse cellular processes, including proliferation and motility; however, it is also implicated in the development of various human cancers [1]. A number of signaling pathways have been identified through which EGFR may regulate rearrangement of actin cytoskeleton, such as activation of phospholipase Cγ1 [2] and Rho GTPases [3,4]. It has been well established that EGF may also signal to actin cytoskeleton via Src tyrosine kinase [5-7]. Recently, the Frank-ter Haar syndrome protein Tks4/HOFI/SH3PXD2B/fad49 (tyrosine kinase substrate with four SH3 domains / homolog of FISH / SH3 and PX domain-containing protein 2B / factor for adipocyte differentiation 49, hereafter termed Tks4) has emerged as a candidate scaffold molecule that has the capability to regulate actin cytoskeleton via Src and EGFR [8,9]. In addition, Tks4 was shown to play an important role in the formation of functional podosomes [10], production of reactive oxygen species (ROS) by tumor cells [11-13], and in the differentiation of white adipose tissue [14].

A close homolog of Tks4 is Tks5/FISH that was first identified as a Src substrate containing one PX and five SH3 domains [15]. Tks5 was shown to be localized at the podosomes of Src-transformed cells and associated with some members of the ADAM metalloprotease family [16]. Later, Tks5 was found to be expressed in podosomes in invasive cancer cells. In addition, Tks5 expression was required for protease-driven matrigel invasion in human cancer cells [17]. In this process Nck adaptor proteins, Nck1 and Nck2, seem to link Tks5 to invadopodia actin regulation and extracellular matrix degradation [18]. Very recently, Tks5 has been shown to be required for migration of neural crest cell during development of zebrafish embryos [19].

In the present study we have investigated the involvement of Tks5 in the EGF signaling pathway. Here we show that upon EGF stimulation of A431 or COS7 cells Tks5 is tyrosine phosphorylated. Using specific kinase inhibitors, we demonstrate that EGF-dependent tyrosine phosphorylation of Tks5 is catalyzed by Src tyrosine kinase. Interestingly, challenge of cells with EGF results in plasma membrane translocation of the scaffold protein. In addition, treatment of cells with LY294002 or BKM120, inhibitors of PI 3-kinase, or mutation of the PX domain reduces tyrosine phosphorylation and membrane translocation of Tks5. Our results suggest that Tks5 as a novel component of the EGF signaling pathway.

## Results

### Tks5 is tyrosine phosphorylated in response to EGF

**Figure 1:**
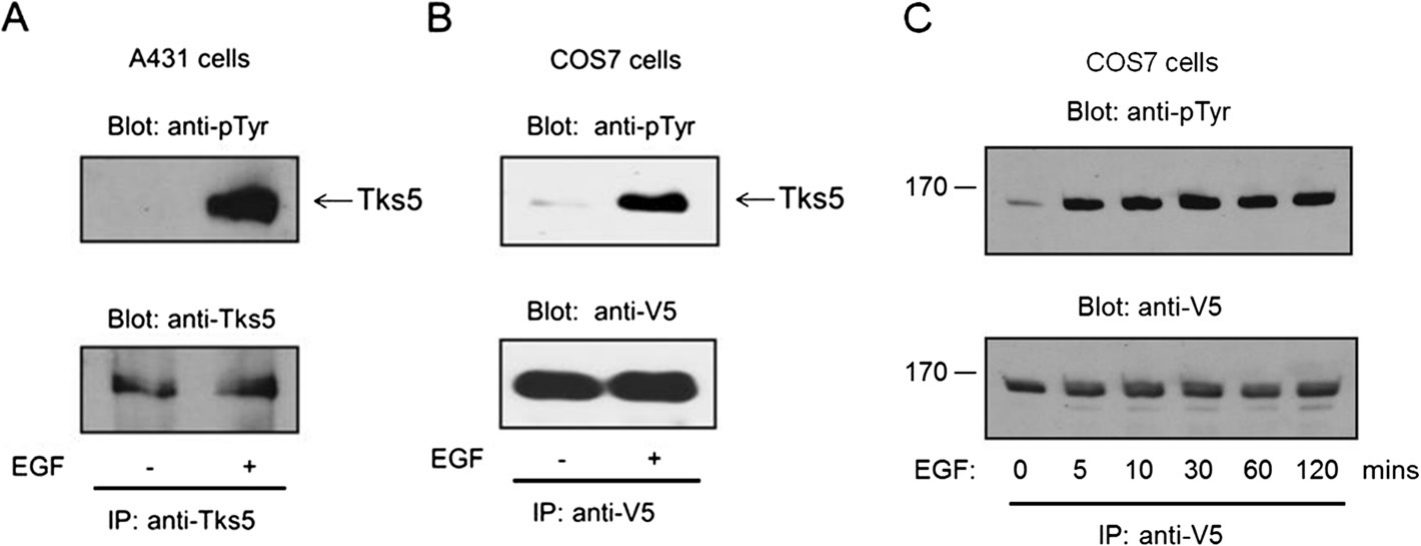
**Tks5 is tyrosine phosphorylated upon EGF treatment of cells**. **(A)** Serum-starved A431 cells were stimulated with EGF (50 ng/ml) for 10 min, and then endogenous Tks5 was immunoprecipitated (IP) with a polyclonal anti-Tks5 antibody. After SDS-PAGE and transfer to nitrocellulose, samples were analysed by anti-phosphotyrosine and anti-Tks5 antibodies. **(B)** Wild type, V5 epitope-tagged Tks5 was transiently expressed in COS7 cells and then serum-starved cells were stimulated with EGF (50 ng/ml) for 10 min or left untreated. Cell lysates were immunoprecipitated with anti-V5 antibody and the immunoprecipitates were immunoblotted with anti-phosphotyrosine and anti-V5 antibodies. **(C)** V5 epitope-tagged Tks5 was transiently expressed in COS7 cells and then serum-starved cells were stimulated with EGF (50 ng/ml) as indicated. Cell lysates were immunoprecipitated with anti-V5 antibody and the immunoprecipitates were immunoblotted with anti-phosphotyrosine and anti-V5 antibodies. These results are representative of three experiments.

Recently, we have shown that Tks4 is implicated in EGF-dependent regulation of actin cytoskeleton [8,9], therefore, we have asked if Tks5, the homolog of Tks4, is also involved directly in the EGF signaling pathway. Serum-starved A431 cells were stimulated with EGF for 10 min or left untreated and then endogenous Tks5 was immunoprecipitated with a polyclonal anti-Tks5 antibody. As seen in Figure 1A, Tks5 is subject of tyrosine phosphorylation in response to EGF stimulation. To confirm the involvement of Tks5 in the EGF signaling pathway in another cell system, V5 epitope-tagged Tks5 was transiently expressed in COS7 cells, then after EGF stimulation Tks5 was immunoprecipitated with an anti-V5 antibody. Figure 1B demonstrates that in this system V5-Tks5 is also tyrosine phosphorylated upon EGF treatment. These results suggest that Tks5 is involved in the EGF signaling pathway.

In 1998, *Lock et al.* investigated if growth factor stimulation resulted in tyrosine phsophorylation of Tks5/FISH [15]. Testing a variety of stimuli they found that treatment of Rat1 fibroblasts with PDGF, LPA, and bradykinin increased the tyrosine phosphorylation of Tks5/FISH. Interestingly, the kinetics of phosphorylation was quite slow in response to PDGF, reaching maximal intensity 2 h after stimulation [15]. Therefore, we measured the time course of tyrosine phosphorylation of Tks5 in response to EGF. V5-Tks5 was transiently expressed in COS7 cells and they were stimulated with EGF for the indicated time periods. Figure 1C demonstrates that the level of phosphorylation reaches its maximum after 5 minutes and this intensity is almost unchanged over the 2 h time period.

### Phosphorylation of Tks5 requires Src kinase

**Figure 2:**
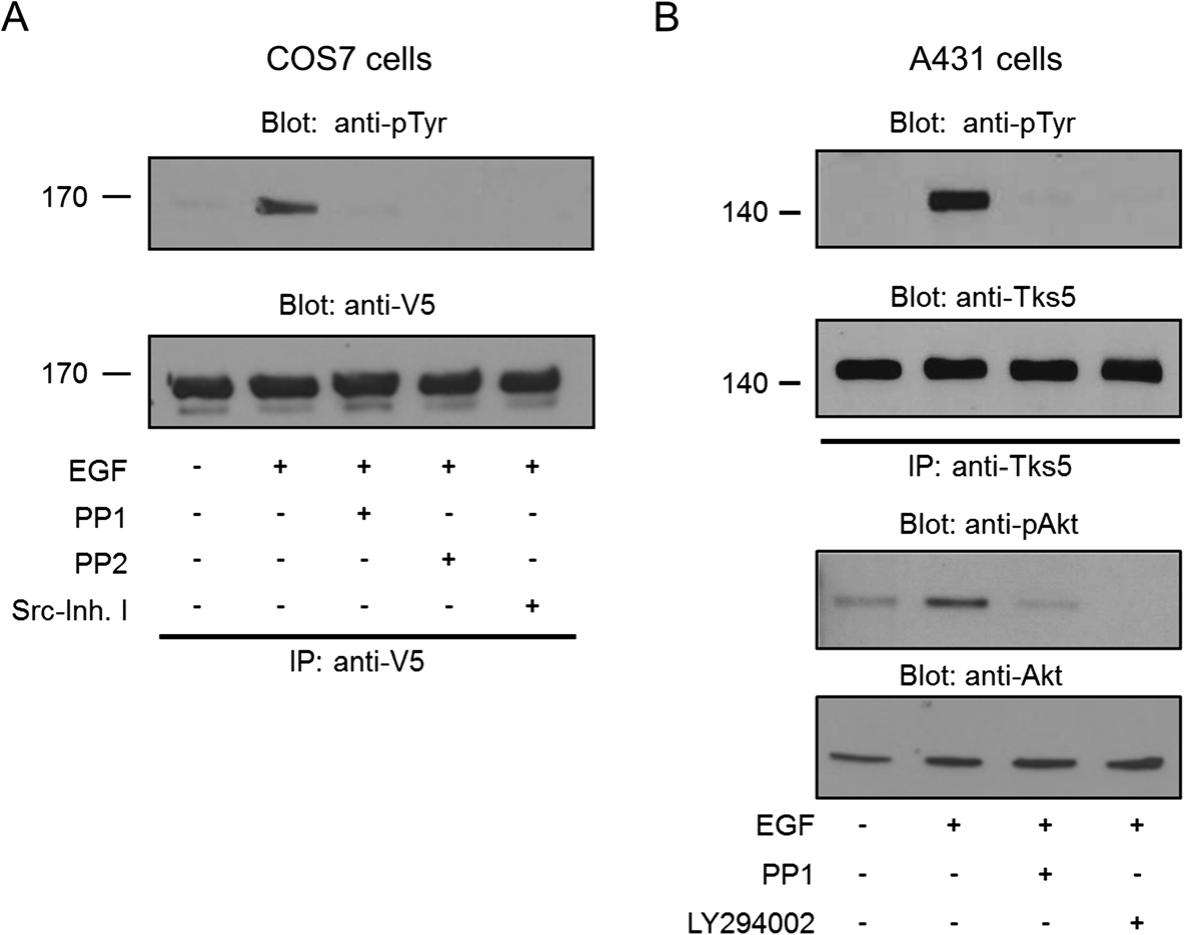
**Phosphorylation of Tks5 upon EGF stimulation requires Src. (A)** COS7 cells were transiently transfected with V5-Tks5 construct and after overnight serum-starvation cells were stimulated with EGF or left untreated. Prior to stimulation, the cells were pretreated with the Src kinase inhibitors as indicated. Tks5 was then immunoprecipitated with anti-V5 antibody and subjected to anti-phosphotyrosine and anti-V5 immunoblots. **(B)** Serum-starved A431 cells were stimulated with EGF (50 ng/ml) for 10 min. Prior to stimulation, the cells were pretreated with the Src kinase inhibitor PP1 and the PI 3-kinase inhibitor LY294002. Endogenous Tks5 was immunoprecipitated (IP) with a polyclonal anti-Tks5 antibody. After SDS-PAGE and transfer to nitrocellulose, samples were analysed by anti-phosphotyrosine and anti-Tks5 antibodies. Cell lysates were also probed with anti-pAKT (Ser473) or anti-Akt antibodies. These results are typical of at least three experiments.

Considering that both Tks4 and its close kin Tks5 are prominent substrates of the Src tyrosine kinase implicated in podosome formation [10,15-17], we supposed that tyrosine kinase Src may be responsible for Tks5 phosphorylation upon EGF stimulation. To challenge our hypothesis, COS7 cells expressing V5-Tks5 were pre-treated with three specific inhibitors of Src, PP1, PP2, and Src kinase inhibitor I, respectively, and following EGF treatment V5-Tks5 was immunoprecipitated and subjected to anti-phosphotyrosine immunoblot. All of the inhibitors markedly decreased tyrosine phosphorylation of Tks5, reflecting the Src kinase is responsible for Tks5 phosphorylation upon EGF stimulation (Figure 2A). To prove that PP1 can also block the tyrosine phosphorylation of the endogenous Tks5, Tks5 was immunoprecipitated from lysates of EGF-treated A431 cells. As seen in Figure 2B, PP1 was capable of inhibiting the EGF-induced tyrosine phosphorylation of Tks5.

### PX domain contributes to the proper phosphorylation of Tks5

**Figure 3:**
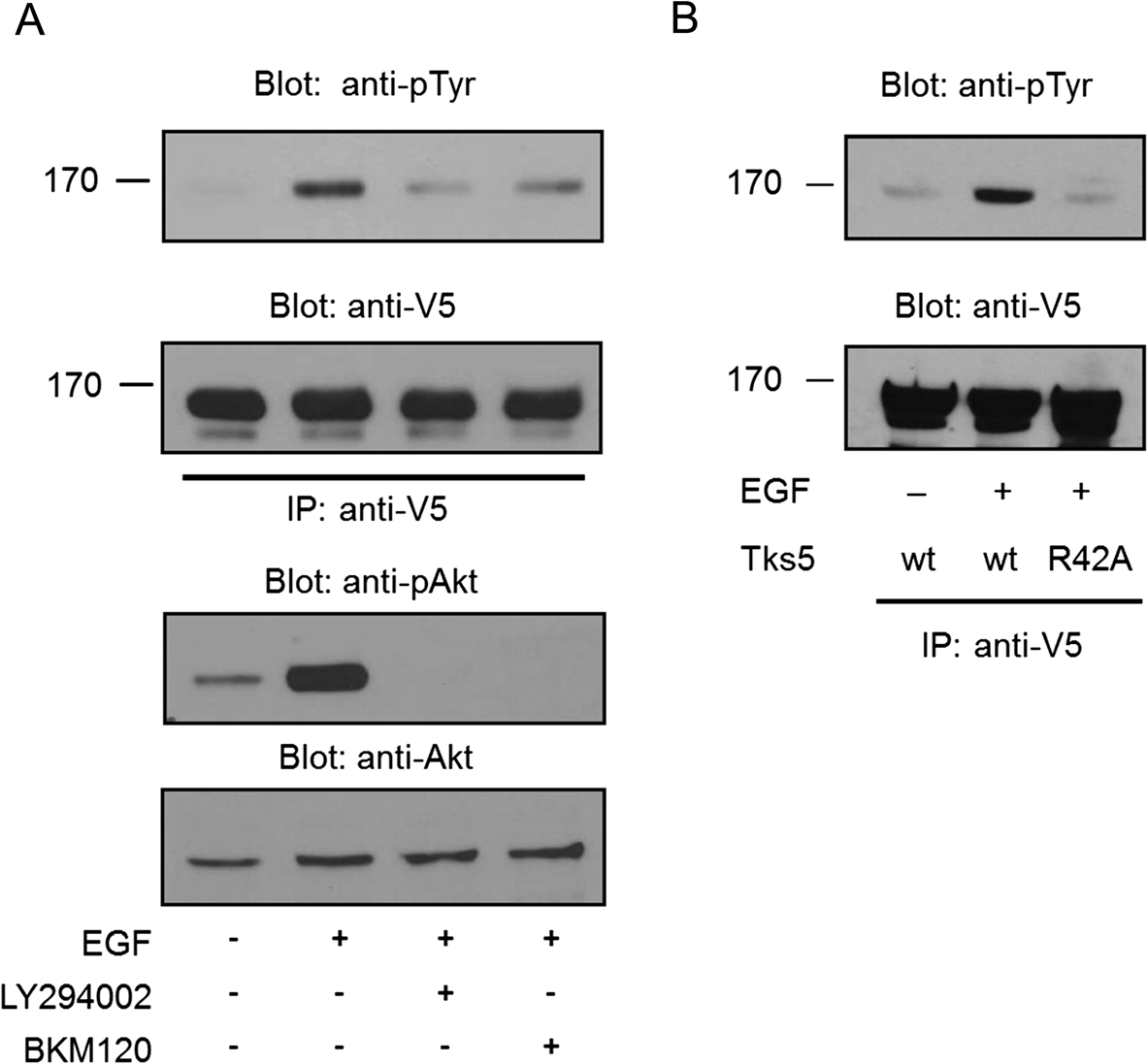
**PI 3-kinase and intact PX domain are required for tyrosine phosphorylation of Tks5. (A)** COS7 cells were transiently transfected with V5-Tks5 and after serum-starvation cells were stimulated with EGF or left untreated. Prior to stimulation the cells were treated with the PI 3-kinase inhibitors LY294002 and BKM120, respectively. Tks5 proteins were immunoprecipitated with anti-V5 antibody and subjected to anti-phosphotyrosine and anti-V5 immunoblots. Cell lysates were also probed with anti-pAKT (Ser473) or anti-Akt antibodies. **(B)** COS7 cells were transiently transfected with the V5-Tks5^R42A^ construct and challenged with EGF. Cell lysates were then subjected to immunoprecipitation with anti-V5 antibody. Bound proteins were separated by SDS-PAGE, transferred to nitrocellulose, and probed with anti-phosphotyrosine and anti-V5 antibodies. These results are typical of at least three experiments.

The family of Tks proteins possesses a Phox homology (PX) domain which can bind specific membrane lipids, such as PtdIns(3)P and PtdIns(3,4), and is implicated in the appropriate cellular localization of Tks4 and Tks5 [9,10,15-17]. Therefore, we asked first if activation of PI 3-kinase producing PtdIns(3)P and PtdIns(3,4) is required for Tks5 phosphorylation. V5 epitope-tagged, wild type Tks5 was transiently expressed in COS7 cells, cells were pretreated with specific PI 3-kinase inhibitors, LY294002 or BKM120, respectively, and then they were stimulated with EGF or left untreated. As shown in Figure 3A, EGF-dependent phosphorylation of Tks5 could be inhibited by the addition of both specific inhibitors. To verify that the specific PI 3-kinase inhibitors really inhibited the enzyme, anti-phospho-Akt immunoblot was preformed from cell lysates. Figure 2B and Figure 3A demonstrate that PI 3-kinase inhibitors blocked effectively the tyrosine phosphorylation of either the endogenous or the V5 epitope-tagged Tks5.

To confirm that the intact PX domain is instrumental for the adequate tyrosine phosphorylation, a point mutation was introduced into the PX domain of Tks5 changing its conserved arginine 42 to alanine, as described earlier [16,20]. Similar mutation was identified in the structure of Tks4 PX domain leading to the change of the highly conserved arginine 43 to triptophan which resulted in the development of Frank-ter Haar syndrome [21]. Latter mutation was predicted to abolish binding to phosphoinositides [21]. Figure 3B shows that the mutation R42A in the PX domain considerably reduced the tyrosine phosphorylation of Tks5. Collectively, these findings suggest that the lipid products of PI 3-kinase and their binding target, the PX domain, are instrumental for the proper tyrosine phosphorylation of Tks5.

### EGF induces translocation of Tks5 to the plasma membrane

**Figure 4:**
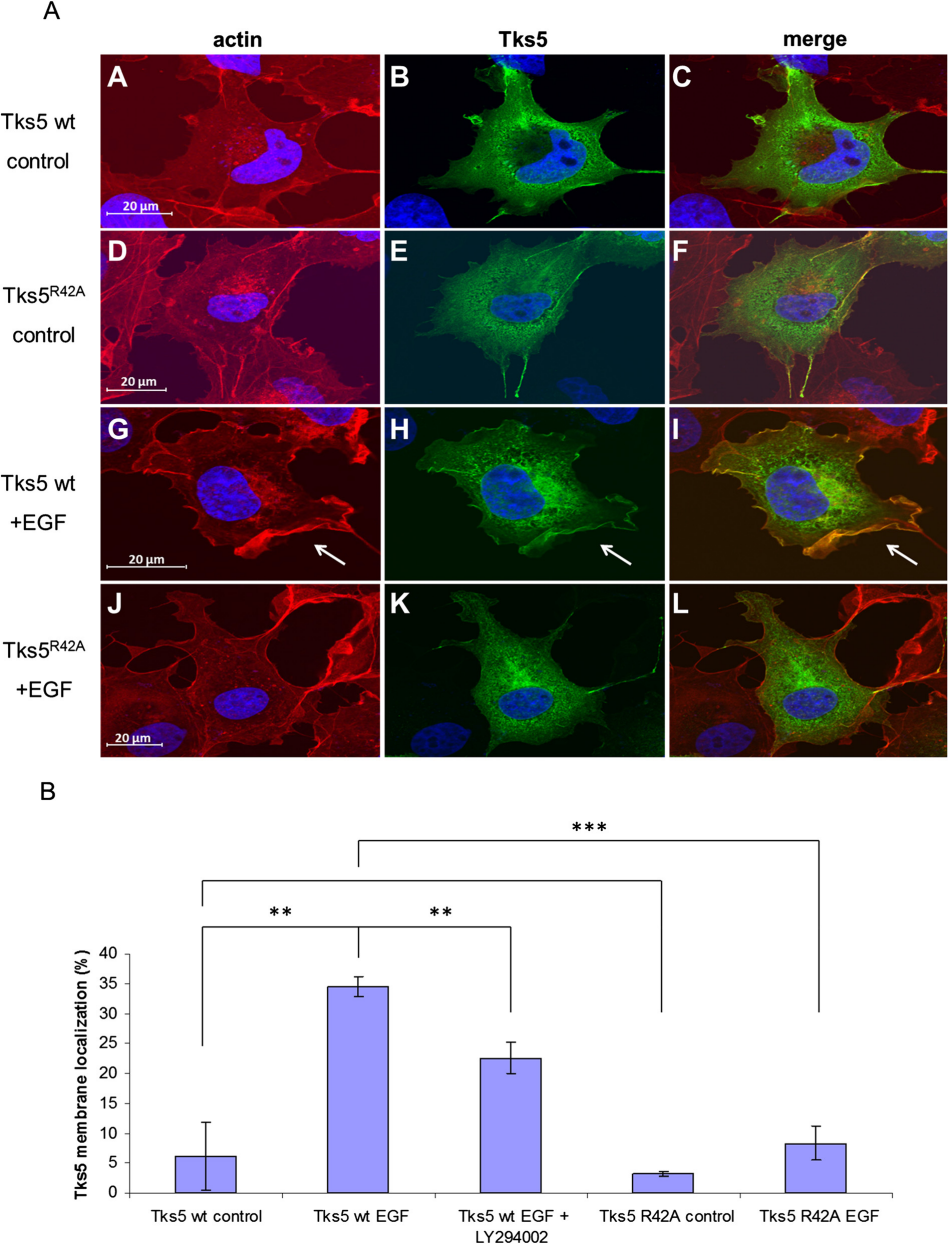
**EGF induces membrane translocation of Tks5 in response to EGF treatment. (A)** COS7 cells were transiently transfected with V5-Tks5 and after serum-starvation cells were stimulated with EGF **(G, H, I)** or left untreated **(A, B, C)**. V5-Tks5^R42A^ construct was also transiently expressed in the cells and after serum-starvation they were treated with EGF **(J, K, L)** or left untreated **(D, E, F)**. Cells were then fixed and processed for immunofluorescence. Subcellular localization of Tks5 was detected using V5-specific monoclonal antibody (B, E, H, and K). To visualize membrane ruffles, cells were also stained with TRITC-phalloidin **(A, D, G, and J)**. Arrows indicate Tks5 present at the plasma membrane. The scale bar represents 20 μm. **(B)** Quantitative analysis of Tks5 translocation upon EGF treatment. COS7 cells were transiently transfected with V5-Tks5 and V5-Tks5^R42A^ constructs and after serum-starvation cells were stimulated with EGF or left untreated. Prior to stimulation a part of the cells expressing V5-Tks5 were treated with the PI 3-kinase inhibitor LY294002 as indicated. The percentage of cells translocated to the plasma membrane under different conditions was quantified by an observer who was blinded to cell treatment status (n = 100 cells for each group per experiment). Error bars represent the Standard Error of the Mean, SEM. ***: p < 0.001, **: p < 0.005

Since the lipid products of PI 3-kinase may recruit Tks5 from the cytosol to the plasma membrane, therefore, we investigated the intracellular rearrangement of Tks5 upon EGF stimulation by confocal microscopy. To this end, V5 epitope-tagged Tks5 was expressed in COS7 cells. In quiescent cells, Tks5 showed a uniform cytoplasmic distribution, with membrane localization in only a low proportion of cells (approx. 6%, Figure 4B). In response to EGF, Tks5 was seen to be translocated to membrane ruffles in approximately 35% of cells (Figure 4). However, when cells were pre-treated with LY294002, membrane translocation of Tks5 was somewhat inhibited, suggested that the lipid products generated by PI 3-kinase upon EGF treatment mediate, at least partially, Tks5 recruitment to the plasma membrane (Figure 4B). Tks5 mutant carrying the point mutation R42A in the PX domain was also tested. While in serum-starved cells membrane localization of the mutant Tks5 was not significantly different from that of the wild type protein, EGF treatment did not induce significant membrane translocation of Tks5 R42A. It is worth to note that although we did not see any difference in the expression levels of wild type and PX domain mutant proteins when they were immunoprecipitated from lysates of COS7 cells, pictures of confocal microscopy suggest that in some of the cells expressing the mutant protein, Tks5 R42A is concentrated next to the nuclei in so called aggresomes (data not shown), reflecting that, similar to the PX domain mutant Tks4, a fraction of mutant Tks5 is likely misfolded [9]. Taken together, these results suggest that EGF regulates Tks5 membrane translocation and this process requires an intact PX domain and the activity of PI 3-kinase.

## Discussion

Recently, we have shown in two studies that Tks4, a kin of Tks5, plays an important role in EGF signaling, regulating the rearrangement of actin cytoskeleton [8,9]. Therefore, in this paper we have investigated if Tks5 could also contribute to EGF signaling. Upon short-term EGF treatment, both endogenous and overexpressed Tks5 were seen to be tyrosine phosphorylated (Figure 1). Although Tks5 was reported earlier to be phosphorylated in Rat1 fibroblasts upon PDGF stimulation, the kinetics of phosphorylation was quite slow, reaching maximal intensity 2 h after stimulation [15]. Therefore, we evaluated V5-Tks5 tyrosine phosphorylation after different intervals of EGF stimulation in COS7 cells. As shown in Figure 1C, level of tyrosine phosphorylation of Tks5 reaches its maximum after 5 minutes and its intensity is almost unchanged over the 2 h time period. This later time course of Tks5 phosphorylation resembles better other proteins seen to be phosphorylated in response to growth factors [22-24].

It has been well established that both Tks4 and Tks5 are prominent substrates of the Src tyrosine kinase [8-10,15-17]. Therefore, it is not surprising that Src tyrosine kinase is responsible for Tks5 phosphorylation upon EGF stimulation (Figure 2). However, the mechanism by which Src phosphorylates the scaffold proteins may differ. In the case of Tks4, stable association of Tks4 with Src was detected in cells over-expressing the kinase [8]. In COS7 cells, upon EGF treatment, inducible interaction was revealed between Tks4 and Src [9]. Conversely, we were not able to detect any kind of association between Src and Tks5 in COS7 cells challenged with EGF (data not shown). These findings suggest that the interaction of Tks4 with Src is likely to be more stable than that of Tks5.

Very recently, we have shown that Tks4 forms a complex with the EGF receptor upon EGF stimulation of cells [9]. Since the interaction of Src kinase with the EGF receptor has been well established, we propose that Src serves as an adaptor molecule which bridges between the EGFR and Tks4. Indeed, we were able to detect an inducible interaction between Tks4 and Src. In addition, expression of SH2 or SH3-deleted mutants of Src in the cells prevented the interaction of Tks4 with EGFR [9]. In contrast, we were not capable of detecting interaction of Tks5 with either Src or the EGF receptor. This finding suggests that although the general structure of Tks4 and Tks5 is very similar their function and regulation are only partially overlapping.

Protein-lipid interaction is a well-underlined mechanism by which eukaryotic cells regulate membrane recruitment [25]. The family of Tks proteins possesses a Phox homology (PX) domain which can bind specific membrane lipids and is implicated in the appropriate cellular localization of Tks4 and Tks5. The PX domain of both Tks4 and Tks5 shows a very similar binding affinity, the preferred lipids are the lipid products of the PI 3-kinase [8-10,16]. Here we show that the PX domain is instrumental for Tks5 to participate properly in the EGF signaling pathway. Point mutation was introduced into the PX domain of Tks5 changing the conserved arginine 42 to alanine, as described earlier [16]. Intriguingly, this mutant was not able to be phosphorylated on tyrosine residues upon EGF treatment (Figure 3B). Moreover, when cells were pretreated with specific inhibitors of PI 3-kinase, EGF-dependent tyrosine phosphorylation of Tks5 was also markedly inhibited (Figure 2B and 3A). When subcellular localization of Tks5 was analyzed by confocal microscopy in EGF treated cells, a significant fraction of Tks5 was seen to be translocated from the cytoplasm to the plasma membrane (Figure 4). This effect was partially prevented by addition of PI 3-kinase inhibitor LY294002. Intriguingly, when an inactivating point mutation (R42A) was introduced into in the PX domain, Tks5 lost its capability to translocate to the plasma membrane upon EGF challenge (Figure 4).

Taken together, we propose that Tks5 is recruited to the plasma membrane via its PX domain in response to EGF treatment. This is reflected in the fact that mutation of one of the conserved arginins in the PX domain known to be essential for lipid binding strongly inhibited the membrane translocation of Tks5. In addition, we found that this Tks5 mutant did not become phosphorylated in EGF-treated cells, suggesting that it could not get in proximity with the membrane-associated Src kinase. Furthermore, membrane translocation of Tks5 requires the activity of PI 3-kinase which is responsible for generating inositol-phospholipids to recruit the PX domain. We have to note that unlike Tks5 Tks4 seems to be recruited to the plasma membrane through at least two independent sites [9]. These are the PX domain which binds the lipid products of PI 3-kinase, and the tyrosine kinase Src which can form an inducible complex with Tks4 at the plasma membrane linking Tks4 to the activated EGF receptor. It is not unique that a regulatory protein requires two independent sites for membrane translocation. For example, the guanine nucleotide exchange factor Sos is recruited to the membrane through interactions with the SH3 domains of adaptor protein Grb2, while its PH domain binds certain phospholipids, such as lipid products of PI 3-kinase or phosphatidic acid [26]. Further experiments will be necessary in the future to reveal why the regulation of Tks5 differs significantly from that of Tks4.

## Conclusion

This study has shown that the scaffold protein Tks5 is a player in the EGF signaling pathway.

It seems that upon EGF treatment, Tks5 is tyrosine phosphorylated within minutes and the level of phosphorylation is sustained for at least 2 hours. We have proved that the tyrosine kinase Src is responsible for the phosphorylation. In addition, the lipid products of PI 3-kinase and the PX domain of Tks5 are instrumental for the EGF-dependent membrane translocation of the scaffold protein. Further experiments will be required to establish the physiological role of Tks5 in the EGF signaling.

## Materials and methods

### Antibodies, constructs and reagents

Antibody against phosphotyrosine residues (clone 4G10, 05–321) was obtained from Millipore (Billerica, MA). Antibody against the V5 epitope (R96025) was ordered from Invitrogen (Carlsbad, CA). Antibodies against pAKT1/2/3 (sc-33437), AKT (sc-5298), and Tks5 (Fish, M-300, sc-30122) were purchased from Santa Cruz Biotechnology, Inc. (Santa Cruz, CA). Antibody against Src (2109) was obtained from Cell Signaling Technology (Beverly, MA). Alexa Fluor 488 rabbit anti-mouse (A11059) antibody was purchased from Invitrogen (Carlsbad, CA). V5-Tks5^R42A^ mutant was generated using the QuickChange Site-Directed Mutagenesis Kit (Stratagene, La Jolla, CA). Stock solutions of epidermal growth factor (EGF, Sigma-Aldrich), PP1 (Biomol, Hamburg, Germany), PP2, Src kinase inhibitor I, BKM120 (Santa Cruz Biotechnology, Inc.) and LY294002 (Merck, Darmstadt, Germany) were prepared according to the manufacturer’s instructions.

### Cell lines, transfection and stimulation

A431 and COS7 cells were purchased from American Type Culture Collection and maintained in Dulbecco’s modified Eagle’s medium (DMEM, Invitrogen) supplemented with 10% Foetal Bovine Serum, penicillin (100 units/ml), streptomycin (100 μg/ml) and L-Glutamine (2 mM). COS7 cells were transiently transfected with Lipofectamine (Invitrogen) according to the manufacturer’s instructions. For stimulation, cells were serum-starved overnight and stimulated with EGF at 50 ng/ml for 10 min. Alternatively, cells were pre-treated with the PI 3-kinase inhibitors (LY294002 at 20 μM and BKM120 at 5 μM) or the Src inhibitors (PP1 at 10 μM, PP2 at 10 μM, and Src kinase 2inhibitor I at 5 μM) for 60 min and then stimulated with EGF as above.

### Confocal microscopy

COS7 cells plated on glass cover slips were transiently transfected with V5-Tks5 or V5-Tks5^R42A^ constructs using Lipofectamine and serum-starved overnight. Cells were pretreated with 20 μM LY294002 for 60 minutes and then treated with 50 ng/ml EGF for 10 minutes. After treatment cells were fixed in 4% paraformaldehyde-PBS for 15 minutes, permeabilized in 0.2% Triton X-100 in PBS for 5 minutes, and blocked with 1% BSA in PBS for 20 minutes. First, the cells were then incubated with TRITC-phalloidin (Sigma-Aldrich) at a final concentration of 0.1 g/ml for 20 min. After careful washing, anti-V5 antibody was applied in 1:1000 dilution for 30 minutes. After washing with PBS the samples were incubated with Alexa Fluor 488 labeled anti-mouse secondary antibody for 30 minutes. After 40 minutes of washing with PBS cover slips were mounted onto slides in a 100 mM Tris–HCl buffer, pH 8.5, containing 10% Mowiol 4–88 (Calbiochem), 25% glycerol, and 2.5% 1,4-diazobicyclo-[2.2.2]octane (DABCO, Sigma-Aldrich). Tks5 membrane-localization was quantified by counting at least 100 cell/sample. Microscopy was performed on a Zeiss LSM 710 confocal microscope.

### Statistics

All quantitative results are presented as the mean and s.d. of (at least 3) independent experiments. Statistical differences between the groups of data were analyzed by Student’s t-test.

## Competing interests

The authors declare that they have no competing interests.

## Author contribution

A.F., G.B, and S.P. carried out experimental design and most of the experimental work. L.B wrote the manuscript. M.G. and L.B. supervised the project. Z.P. prepared some of the cDNA constructs. All authors read and approved the final manuscript.

## References

[B1] Hynes NE Lane HA ERBB receptors and cancer: the complexity of targeted inhibitors Nat Rev Cancer 2005 5 5 341 354 10.1038/nrc1609 15864276

[B2] Diakonova M Payrastre B Van Velzen AG Hage WJ van Bergen en Henegouwen PM Boonstra J Cremers FF Humbel BM Epidermal growth factor induces rapid and transient association of phospholipase C-gamma 1 with EGF-receptor and filamentous actin at membrane ruffles of A431 cells J Cell Sci 1995 108 Pt 6 2499 2509 7673364 10.1242/jcs.108.6.2499

[B3] Ridley AJ Paterson HF Johnston CL Diekmann D Hall A The small GTP-binding protein rac regulates growth factor-induced membrane ruffling Cell 1992 70 3 401 410 10.1016/0092-8674(92)90164-8 1643658

[B4] Tamas P Solti Z Bauer P Illes A Sipeki S Bauer A Farago A Downward J Buday L Mechanism of epidermal growth factor regulation of Vav2, a guanine nucleotide exchange factor for Rac The Journal of biological chemistry 2003 278 7 5163 5171 10.1074/jbc.M207555200 12454019

[B5] Belsches AP Haskell MD Parsons SJ Role of c-Src tyrosine kinase in EGF-induced mitogenesis Front Biosci 1997 2 d501 d518 9331427 10.2741/a208

[B6] Neumann-Giesen C Fernow I Amaddii M Tikkanen R Role of EGF-induced tyrosine phosphorylation of reggie-1/flotillin-2 in cell spreading and signaling to the actin cytoskeleton J Cell Sci 2007 120 Pt 3 395 406 17213334 10.1242/jcs.03336

[B7] Itoh RE Kiyokawa E Aoki K Nishioka T Akiyama T Matsuda M Phosphorylation and activation of the Rac1 and Cdc42 GEF Asef in A431 cells stimulated by EGF J Cell Sci 2008 121 Pt 16 2635 2642 18653540 10.1242/jcs.028647

[B8] Lanyi A Barath M Peterfi Z Bogel G Orient A Simon T Petrovszki E Kis-Toth K Sirokmany G Rajnavolgyi E The Homolog of the Five SH3-Domain Protein (HOFI/SH3PXD2B) Regulates Lamellipodia Formation and Cell Spreading PloS one 2011 6 8 e23653 10.1371/journal.pone.0023653 21886807 PMC3160312

[B9] Bogel G Gujdar A Geiszt M Lanyi A Fekete A Sipeki S Downward J Buday L Frank-ter Haar syndrome protein Tks4 regulates EGF-dependent cell migration The Journal of biological chemistry 2012 287 37 31321 31329 10.1074/jbc.M111.324897 22829589 PMC3438961

[B10] Buschman MD Bromann PA Cejudo-Martin P Wen F Pass I Courtneidge SA The novel adaptor protein Tks4 (SH3PXD2B) is required for functional podosome formation Mol Biol Cell 2009 20 5 1302 1311 10.1091/mbc.E08-09-0949 19144821 PMC2649273

[B11] Gianni D Diaz B Taulet N Fowler B Courtneidge SA Bokoch GM Novel p47(phox)-related organizers regulate localized NADPH oxidase 1 (Nox1) activity Sci Signal 2009 2 88 ra54 10.1126/scisignal.2000370 19755710 PMC2850287

[B12] Gianni D Taulet N DerMardirossian C Bokoch GM c-Src-mediated phosphorylation of NoxA1 and Tks4 induces the reactive oxygen species (ROS)-dependent formation of functional invadopodia in human colon cancer cells Mol Biol Cell 2010 21 23 4287 4298 10.1091/mbc.E10-08-0685 20943948 PMC2993755

[B13] Gianni D DerMardirossian C Bokoch GM Direct interaction between Tks proteins and the N-terminal proline-rich region (PRR) of NoxA1 mediates Nox1-dependent ROS generation Eur J Cell Biol 2011 90 2–3 164 171 20609497 10.1016/j.ejcb.2010.05.007 PMC3013238

[B14] Hishida T Eguchi T Osada S Nishizuka M Imagawa M A novel gene, fad49, plays a crucial role in the immediate early stage of adipocyte differentiation via involvement in mitotic clonal expansion Febs J 2008 275 22 5576 5588 10.1111/j.1742-4658.2008.06682.x 18959745

[B15] Lock P Abram CL Gibson T Courtneidge SA A new method for isolating tyrosine kinase substrates used to identify fish, an SH3 and PX domain-containing protein, and Src substrate The EMBO journal 1998 17 15 4346 4357 10.1093/emboj/17.15.4346 9687503 PMC1170768

[B16] Abram CL Seals DF Pass I Salinsky D Maurer L Roth TM Courtneidge SA The adaptor protein fish associates with members of the ADAMs family and localizes to podosomes of Src-transformed cells The Journal of biological chemistry 2003 278 19 16844 16851 10.1074/jbc.M300267200 12615925

[B17] Seals DF Azucena EF Jr Pass I Tesfay L Gordon R Woodrow M Resau JH Courtneidge SA The adaptor protein Tks5/Fish is required for podosome formation and function, and for the protease-driven invasion of cancer cells Cancer Cell 2005 7 2 155 165 10.1016/j.ccr.2005.01.006 15710328

[B18] Stylli SS Stacey TT Verhagen AM Xu SS Pass I Courtneidge SA Lock P Nck adaptor proteins link Tks5 to invadopodia actin regulation and ECM degradation J Cell Sci 2009 122 Pt 15 2727 2740 19596797 10.1242/jcs.046680 PMC2909319

[B19] Murphy DA Diaz B Bromann PA Tsai JH Kawakami Y Maurer J Stewart RA Izpisua-Belmonte JC Courtneidge SA A Src-Tks5 pathway is required for neural crest cell migration during embryonic development PloS one 2011 6 7 e22499 10.1371/journal.pone.0022499 21799874 PMC3143166

[B20] Oikawa T Itoh T Takenawa T Sequential signals toward podosome formation in NIH-src cells The Journal of cell biology 2008 182 1 157 169 10.1083/jcb.200801042 18606851 PMC2447888

[B21] Iqbal Z Cejudo-Martin P De Brouwer A van der Zwaag B Ruiz-Lozano P Scimia MC Lindsey JD Weinreb R Albrecht B Megarbane A Disruption of the podosome adaptor protein TKS4 (SH3PXD2B) causes the skeletal dysplasia, eye, and cardiac abnormalities of Frank-Ter Haar Syndrome Am J Hum Genet 2010 86 2 254 261 10.1016/j.ajhg.2010.01.009 20137777 PMC2820172

[B22] Feng Q Baird D Peng X Wang J Ly T Guan JL Cerione RA Cool-1 functions as an essential regulatory node for EGF receptor- and Src-mediated cell growth Nat Cell Biol 2006 8 9 945 956 10.1038/ncb1453 16892055

[B23] Li S Wang Q Wang Y Chen X Wang Z PLC-gamma1 and Rac1 coregulate EGF-induced cytoskeleton remodeling and cell migration Molecular endocrinology 2009 23 6 901 913 10.1210/me.2008-0368 19264842 PMC5419285

[B24] Samson T Welch C Monaghan-Benson E Hahn KM Burridge K Endogenous RhoG is rapidly activated after epidermal growth factor stimulation through multiple guanine-nucleotide exchange factors Mol Biol Cell 2010 21 9 1629 1642 10.1091/mbc.E09-09-0809 20237158 PMC2861620

[B25] Seet LF Hong W The Phox (PX) domain proteins and membrane traffic Biochim Biophys Acta 2006 1761 8 878 896 10.1016/j.bbalip.2006.04.011 16782399

[B26] Buday L Downward J Many faces of Ras activation Biochim Biophys Acta 2008 1786 2 178 187 18541156 10.1016/j.bbcan.2008.05.001

